# Crystal structures of two 1:2 dihydrate compounds of chloranilic acid with 2-carb­oxy­pyridine and 2-carb­oxy­quinoline

**DOI:** 10.1107/S2056989017015997

**Published:** 2017-11-07

**Authors:** Kazuma Gotoh, Hiroyuki Ishida

**Affiliations:** a Department of Chemistry, Faculty of Science, Okayama University, Okayama 700-8530, Japan

**Keywords:** crystal structure, chloranilic acid, 2-carb­oxy­pyridine, 2-carb­oxy­quinoline, twitterion, disorder, hydrogen bond

## Abstract

The crystal structure of the 1:2 dihydrate compound of chloranilic acid (systematic name: 2,5-di­chloro-3,6-dihy­droxy-1,4-benzo­quinone) with 2-carb­oxy­pyridine (another common name: picolinic acid; systematic name: pyridine-2-carb­oxy­lic acid), namely, 2C_6_H_5.5_NO_2_^0.5+^·C_6_HCl_2_O_4_^−^·2H_2_O, (I), has been determined at 180 K, and the structure of the 1:2 dihydrate compound of chloranilic acid with 2-carb­oxy­quinoline (another common name: quinaldic acid; systematic name: quinoline-2-carb­oxy­lic acid), namely, 2C_10_H_7_NO_2_·C_6_H_2_Cl_2_O_4_·2H_2_O, (II), has been redetermined at 200 K. This determination presents a higher precision crystal structure than the previously published structure [Marfo-Owusu & Thompson (2014 ▸). *X-ray Struct. Anal. Online*, **30**, 55–56]. Compound (I) was analysed as a disordered structure over two states, *viz.* salt and co-crystal. The salt is bis­(2-carb­oxy­pyridinium) chloranilate dihydrate, 2C_6_H_6_NO_2_^+^·C_6_Cl_2_O_4_^2−^·2H_2_O, and the co-crystal is bis­(pyridinium-2-carboxyl­ate) chloranilic acid dihydrate, 2C_6_H_5_NO_2_·C_6_H_2_Cl_2_O_4_·2H_2_O, including zwitterionic 2-carb­oxy­pyridine. In both salt and co-crystal, the water mol­ecule links the chloranilic acid and 2-carb­oxy­pyridine mol­ecules through O—H⋯O and N—H⋯O hydrogen bonds. The 2-carb­oxy­pyridine mol­ecules are connected into a head-to-head inversion dimer by a short O—H⋯O hydrogen bond, in which the H atom is disordered over two positions. Compound (II) is a 1:2 dihydrate co-crystal of chloranilic acid and zwitterionic 2-carb­oxy­quinoline. The water mol­ecule links the chloranilic acid and 2-carb­oxy­quinoline mol­ecules through O—H⋯O hydrogen bonds. The 2-carb­oxy­quinoline mol­ecules are connected into a head-to-tail inversion dimer by a pair of N—H⋯O hydrogen bonds.

## Abstract 2

Crystal structures of hydrogen-bonded 1:2 dihydrate compounds of chloranilic acid with 2-carb­oxy­pyridine (I) and 2-carb­oxy­quinoline (II) have been determined at 180 and 200 K, respectively. The base mol­ecule in (I) is disordered over cationic and twitterionic states, while that in (II) is in a twitterionic form. In each crystal, the three components are linked by O—H⋯O and N—H⋯O hydrogen bonds, forming a layer structure.

## Chemical context   

Chloranilic acid, a dibasic acid with hydrogen-bond donor as well as acceptor groups, appears particularly attractive as a template for generating tightly bound self-assemblies with various pyridine derivatives as well as being a model compound for investigating hydrogen-transfer motions in O—H⋯N and N—H⋯O hydrogen-bond systems (Zaman *et al.*, 2004 ▸; Molčanov & Kojić-Prodić, 2010 ▸; Seliger *et al.*, 2009 ▸; Asaji *et al.* 2010 ▸). Previously, we have prepared three 1:1 compounds of chloranilic acid with 2-, 3- and 4-carb­oxy­pyridine and analysed the crystal structures in order to extend our study on *D*—H⋯*A* hydrogen bonding (*D* = N, O or C; *A* = N, O or Cl) in chloranilic acid–substituted pyridine systems (Gotoh *et al.*, 2006 ▸, 2009 ▸; Tabuchi *et al.*, 2005 ▸). In the present study, we have prepared a 1:2 compound of chloranilic acid with 2-carb­oxy­pyridine and also redetermined the structure of a 1:2 compound of chloranilic acid with 2-carb­oxy­quinoline with higher precision than previously reported structure [Marfo-Owusu & Thompson, 2014 ▸; although the title and text in this reference refer to the 1:1 adduct of chloranilic acid with 2-carb­oxy­qulinone, the reported structure is the 1:2 compound, the same as the present compound (II)]. The crystal structure of the anhydrous 1:2 compound of chloranilic acid with 2-carb­oxy­quinoline was also reported by Marfo-Owusu & Thompson (2016 ▸).
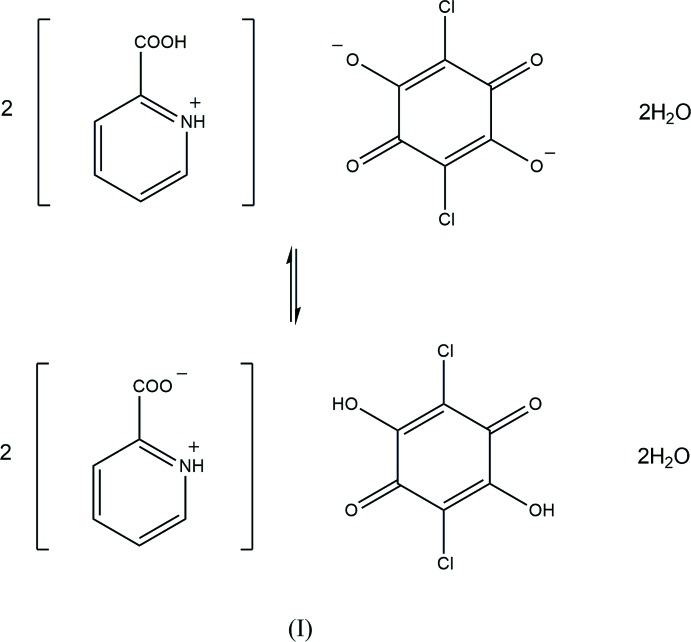
 
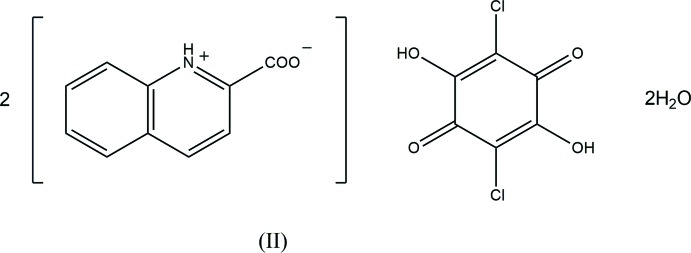


## Structural commentary   

**Figure 1:**
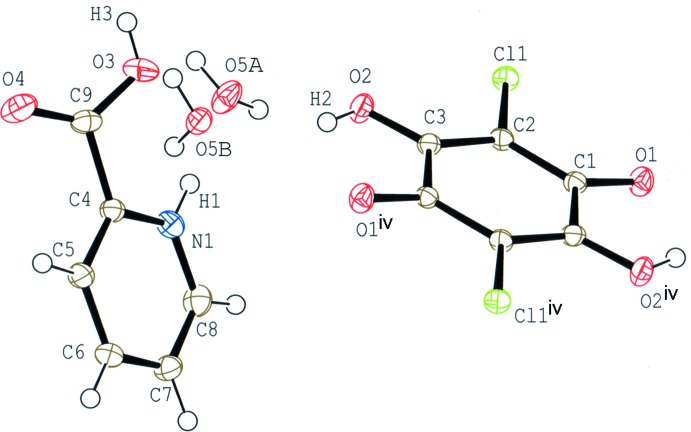
The mol­ecular structure of compound (I), showing the atom-numbering scheme. Displacement ellipsoids of non-H atoms are drawn at the 50% probability level and H atoms are drawn as small spheres of arbitrary radii. The water mol­ecule is disordered over two sites with equally occupancies. Atoms H2 and H3 have site-occupancy factors of 0.5. [Symmetry code: (iv) −*x* + 1, −*y* + 1, −*z* + 1.]

**Figure 2:**
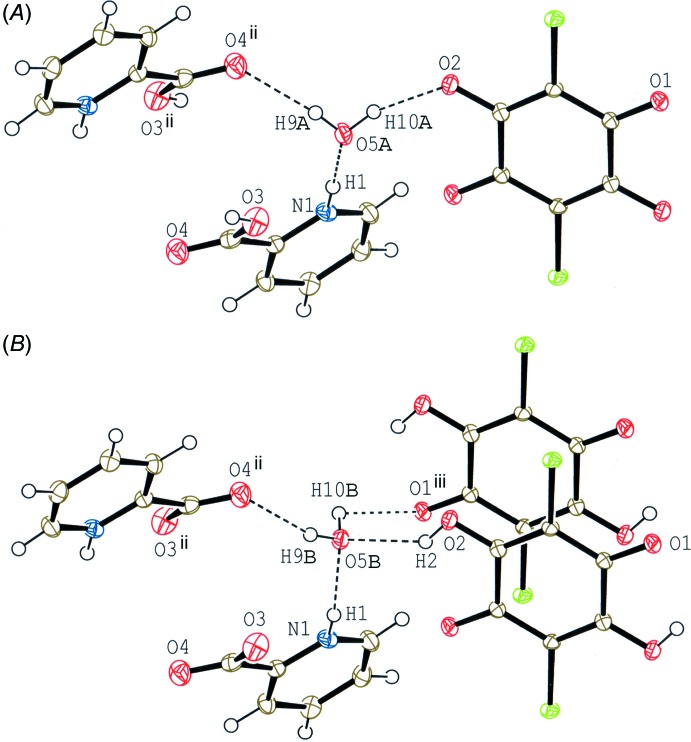
A partial packing diagram of compound (I) around the disordered water mol­ecule in bis­(2-carb­oxy­pyridinium) chloranilate dihydrate (*A*) and bis­(pyridinium-2-carboxyl­ate) chloranilic acid dihydrate (*B*), showing O—H⋯O and N—H⋯O hydrogen bonds (dashed lines). [Symmetry codes: (ii) −*x*, −*y*, −*z* + 1; (iii) −*x* + 1, −*y*, −*z* + 1.]

Compound (I) (Fig. 1 ▸) crystallizes with one-half of a chloranilic acid mol­ecule, which is located on an inversion centre, one 2-carb­oxy­pyridine mol­ecule and one water mol­ecule in the asymmetric unit. In the crystal, the water mol­ecule is disordered over two sites with equal occupancies of 0.5. The occupancies of the H atoms in the chloranilic acid mol­ecule and the carb­oxy group of the 2-carb­oxy­pyridine mol­ecule are also 0.5. The compound is, therefore, considered to be a disordered state over two forms, *viz*. bis­(2-carb­oxy­pyridinium) chloranilate dihydrate, (*A*), and bis­(pyridinium-2-carboxyl­ate) chloranilic acid dihydrate, (*B*), as shown in the scheme and Fig. 2 ▸. In form (*A*), the water mol­ecule acts as one N—H⋯O hydrogen-bond acceptor and two O—H⋯O hydrogen-bond donors (N1—H1⋯O5*A*, O5*A*—O9*A*⋯O4^ii^ and O5*A*—H10*A*⋯O2; symmetry code as in Table 1 ▸), while in form (*B*), the water mol­ecule acts as the acceptor of N—H⋯O and O—H⋯O hydrogen bonds, and as two O—H⋯O hydrogen-bond donors (N1—H1⋯O5*B*, O2—H2⋯O5*B*, O5*B*—H9*B*⋯O4^ii^ and O5*B*—H10*B*⋯O1^iii^; Table 1 ▸). The dihedral angle between the pyridine ring and the carb­oxy plane in the base mol­ecule is 23.32 (15)°.

**Figure 3:**
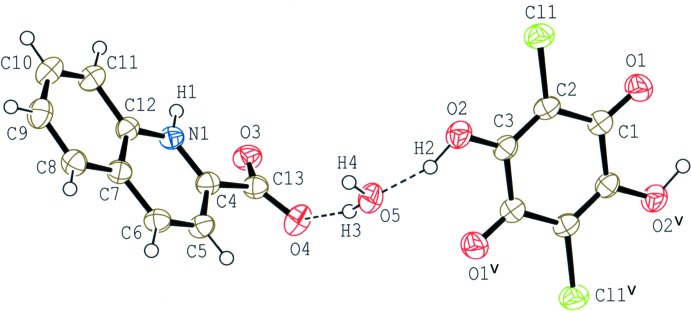
The mol­ecular structure of compound (II), showing the atom-numbering scheme. Displacement ellipsoids of non-H atoms are drawn at the 50% probability level and H atoms are drawn as small spheres of arbitrary radii. O—H⋯O hydrogen bonds are shown as dashed lines. [Symmetry code: (v) −*x*, −*y*, −*z*.]

The asymmetric unit of compound (II) consists of one-half of a chloranilic acid mol­ecule, which is located on an inversion centre, one 2-carb­oxy­quinoline mol­ecule and one water mol­ecule. In the crystal, the 2-carb­oxy­quinoline mol­ecule is in a twitterionic form and no acid–base inter­action involving H-atom transfer between chloranilic acid and 2-carb­oxy­quinoline is observed (Fig. 3 ▸). The dihedral angle between the quinoline ring system and the carboxyl­ate plane in the base mol­ecule is 20.84 (19)°. The water mol­ecule acts as an O—H⋯O hydrogen-bonding bridge between the chloranilic and 2-carb­oxy­quinoline mol­ecules (O2—H2⋯O5 and O5—H3⋯O4; Table 2 ▸).

## Supra­molecular features   

**Figure 4:**
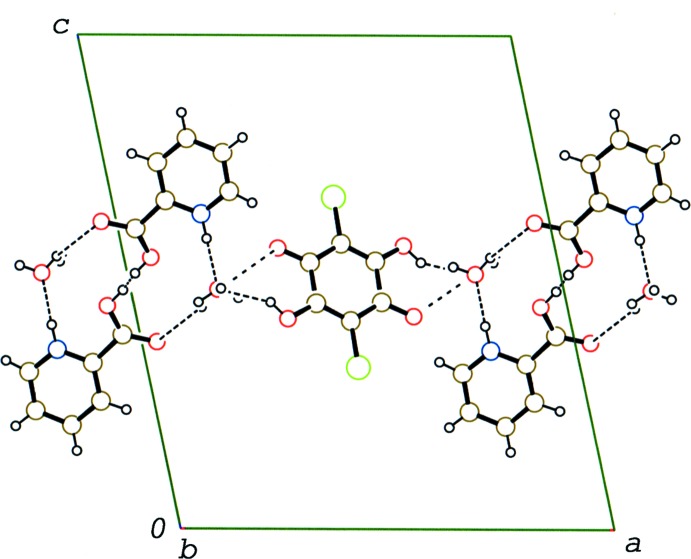
A packing diagram of compound (I) viewed along the *b* axis, showing the layer structure. O—H⋯O and N—H⋯O hydrogen bonds are shown as dashed lines.

In the crystal of compound (I), the 2-carb­oxy­pyridine mol­ecules, which are related by an inversion centre, are linked into a head-to-head dimer *via* a short O—H⋯O hydrogen bond, in which the H atom is disordered over two sites (O3—H3⋯O3^i^; Table 1 ▸), as observed in pyridinium-2-carb­oxy­lic acid pyridinium-2-carboxyl­ate perchlorate (Wang *et al.*, 2015 ▸). The three components are linked *via* the above-mentioned O—H⋯O and N—H⋯O hydrogen bonds together with weak C—H⋯Cl and C—H⋯O hydrogen bonds (C8—H8⋯Cl1^iii^ and C8—H8⋯O1^iii^; Table 1 ▸), forming a layer parallel to the *ab* plane (Fig. 4 ▸). In the layer, the chloranilic acid rings are stacked along the *b* axis through a π–π inter­action [centroid–centroid distance = 3.6851 (7) Å and inter­planar spacing = 3.2118 (4) Å]. The pyridine rings are also stacked along the *b* axis through a π–π inter­action [centroid–centroid distance = 3.6851 (7) Å and inter­planar spacing = 3.4787 (5) Å]. Between the layers, a short Cl⋯Cl contact is observed [Cl1⋯Cl1^v^ = 3.3717 (5) Å; symmetry code: (v) −*x* + 1, *y* − 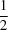, −*z* + 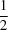].

**Figure 5:**
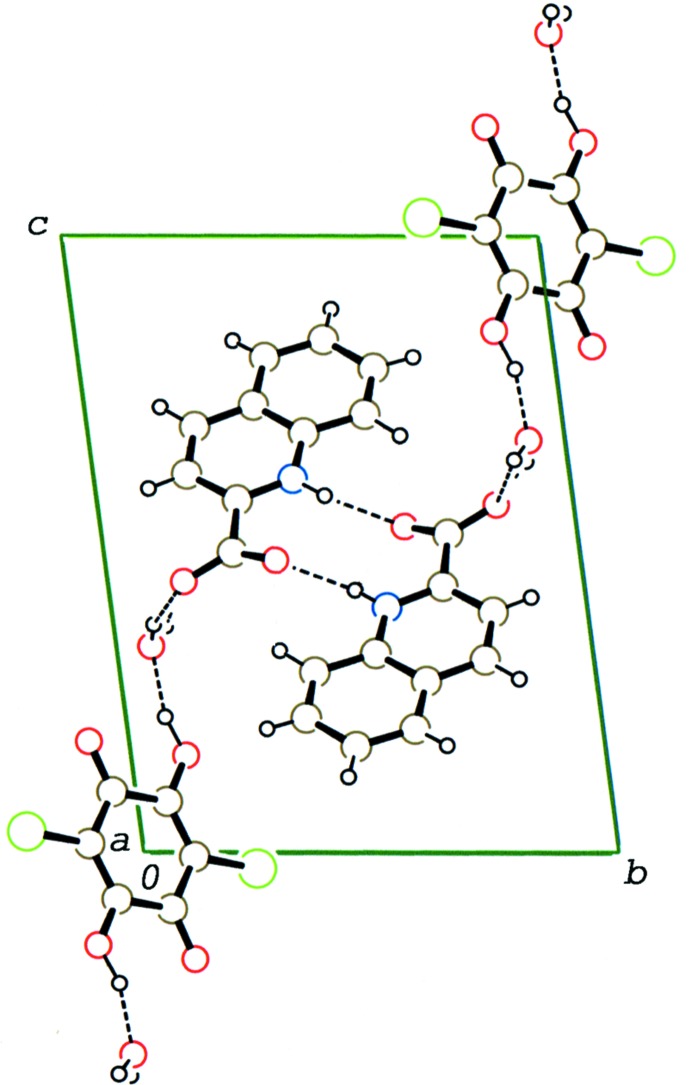
A packing diagram of compound (II) viewed along the *a* axis, showing the layer structure formed by O—H⋯O and N—H⋯O hydrogen bonds (dashed lines).

In the crystal of (II), two adjacent 2-carb­oxy­quinoline mol­ecules, which are related by an inversion centre, form a head-to-tail dimer *via* a pair of N—H⋯O hydrogen bonds (N—H1⋯O3^i^; symmetry code as in Table 2 ▸). The dimers are stacked in a column along the *a* axis through a weak π–π inter­action between the N1/C4–C7/C12 and C7–C12 rings with a centroid–centroid distance of 3.9184 (10) Å. The water mol­ecule links the stacked base mol­ecules related by translation along *a via* O—H⋯O hydrogen bonds [O5—H3⋯O4 and O5—H4⋯O4^ii^; Table 2 ▸] and also links the acid mol­ecule and the two base mol­ecules *via* O—H⋯O hydrogen bonds, forming a layer structure parallel to (0
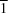
1) as shown in Fig. 5 ▸. No significant short contact between the acid mol­ecules in the layer is observed. Between the layers, a bifurcated C—H⋯(O, O) hydrogen bond (C6—H6⋯O1^iii^ and C6—H6⋯O5^iv^; Table 2 ▸) is observed, through which the 2-carboxy­quinoline mol­ecule is weakly linked with the chloranilic acid and water mol­ecules.

## Database survey   

A search of the Cambridge Structural Database (Version 5.38, last update May 2017; Groom *et al.*, 2016 ▸) for organic co-crystals of pyridinium-2-carboxyl­ate (twitterionic form) gave six structures. For organic co-crystals of quinolinium-2-carboxyl­ate (twitterionic form), eight structures were found.

## Synthesis and crystallization   

Single crystals of compound (I) were obtained by slow evaporation of an aceto­nitrile solution (200 ml) of chloranilic acid (250 mg) with 2-carb­oxy­pridine (310 mg) at room temperature. Single crystals of compound (II) were obtained by slow evaporation from a methanol solution (150 ml) of chloranilic acid (310 mg) with 2-carb­oxy­quinoline (520 mg) at room temperature.

## Refinement   

Crystal data, data collection and structure refinement details are summarized in Table 3 ▸. The water mol­ecule in compound (I) was found to be disordered over two sites in a difference-Fourier map. The occupancies were refined to 0.52 (2) and 0.48 (2) and then they were fixed at 0.5. The H atom in the carb­oxy group of the base mol­ecule was also found in a difference-Fourier map to be disordered between the adjacent carb­oxy groups, which are related by an inversion centre, and the occupancy was set to be 0.5. Since the N-bound H atom refined reasonably with an occupancy of 1, the occupancy of the H atom of the acid mol­ecule was set to be 0.5 to balance the total charge of the compound. All other H atoms were found in a difference-Fourier map. The N-bound H atom was refined freely, while the positions of O-bound H atoms were refined, with O—H = 0.84 (2) Å and *U*_iso_(H) = 1.5*U*_eq_(O). For the water H atoms, distant restraints of H⋯H = 1.37 (4) Å were also applied. C-bound H atoms were positioned geometrically (C—H = 0.95 Å) and were treated as riding with *U*_iso_(H) = 1.2*U*_eq_(C).

All H atoms in compound (II) were found in a difference-Fourier map. The O- and N-bound H atoms in the acid and base mol­ecules were refined freely. The water H atoms were refined with O—H = 0.84 (2) Å. C-bound H atoms were positioned geometrically (C—H = 0.95 Å) and were treated as riding with *U*_iso_(H) = 1.2*U*_eq_(C).

## Supplementary Material

Crystal structure: contains datablock(s) General, I, II. DOI: 10.1107/S2056989017015997/lh5860sup1.cif

Structure factors: contains datablock(s) I. DOI: 10.1107/S2056989017015997/lh5860Isup2.hkl

Structure factors: contains datablock(s) II. DOI: 10.1107/S2056989017015997/lh5860IIsup3.hkl

Supporting information file. DOI: 10.1107/S2056989017015997/lh5860IIsup4.cml

CCDC references: 1583721, 1583720

Additional supporting information:  crystallographic information; 3D view; checkCIF report

## Figures and Tables

**Table 1:** Hydrogen-bond geometry (Å, °) for (I)

*D*—H⋯*A*	*D*—H	H⋯*A*	*D*⋯*A*	*D*—H⋯*A*
N1—H1⋯O5*A*	0.895 (17)	1.843 (17)	2.7380 (19)	179 (2)
N1—H1⋯O5*B*	0.895 (17)	1.907 (17)	2.7417 (18)	154 (2)
O2—H2⋯O5*B*	0.83 (3)	2.12 (3)	2.8111 (19)	141 (3)
O3—H3⋯O3^i^	0.82 (3)	1.62 (4)	2.4352 (14)	174 (4)
O5*A*—H9*A*⋯O4^ii^	0.82 (3)	2.11 (3)	2.927 (2)	170 (3)
O5*B*—H9*B*⋯O4^ii^	0.82 (3)	2.02 (3)	2.8159 (19)	162 (3)
O5*A*—H10*A*⋯O2	0.84 (3)	1.90 (3)	2.6762 (19)	154 (4)
O5*B*—H10*B*⋯O1^iii^	0.85 (2)	2.60 (3)	3.095 (2)	119 (2)
C8—H8⋯Cl1^iii^	0.95	2.78	3.6524 (12)	154
C8—H8⋯O1^iii^	0.95	2.47	3.1871 (14)	132

Symmetry codes: (i) 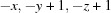; (ii) 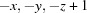; (iii) 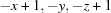.

**Table 2:** Hydrogen-bond geometry (Å, °) for (II)

*D*—H⋯*A*	*D*—H	H⋯*A*	*D*⋯*A*	*D*—H⋯*A*
N1—H1⋯O3^i^	0.88 (2)	1.91 (2)	2.7724 (17)	167 (2)
O2—H2⋯O5	0.98 (3)	1.59 (3)	2.5092 (17)	155 (3)
O5—H3⋯O4	0.82 (2)	2.01 (2)	2.8072 (19)	164 (2)
O5—H4⋯O4^ii^	0.85 (2)	1.82 (2)	2.6632 (19)	171 (2)
C6—H6⋯O1^iii^	0.95	2.54	3.392 (2)	150
C6—H6⋯O5^iv^	0.95	2.47	3.211 (2)	134

Symmetry codes: (i) 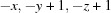; (ii) 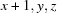; (iii) 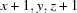; (iv) 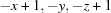.

**Table 3:** Experimental details

	(I)	(II)
Crystal data		
Chemical formula	2C_6_H_5.5_NO_2_^0.5+^·C_6_HCl_2_O_4_^−^·2H_2_O	2C_10_H_7_NO_2_·C_6_H_2_Cl_2_O_4_·2H_2_O
*M*_r_	491.24	591.36
Crystal system, space group	Monoclinic, *P*2_1_/*c*	Triclinic, *P* 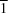
Temperature (K)	180	200
*a*, *b*, *c* (Å)	15.1028 (10), 3.6851 (3), 17.5689 (13)	4.4745 (2), 10.5448 (8), 13.6111 (6)
α, β, γ (°)	90, 101.871 (3), 90	96.652 (4), 94.109 (3), 99.009 (4)
*V* (Å^3^)	956.89 (12)	627.38 (6)
*Z*	2	1
Radiation type	Mo *K*α	Mo *K*α
μ (mm^−1^)	0.40	0.32
Crystal size (mm)	0.35 × 0.18 × 0.13	0.41 × 0.21 × 0.03

Data collection		
Diffractometer	Rigaku R-AXIS RAPIDII	Rigaku R-AXIS RAPIDII
Absorption correction	Numerical (*NUMABS*; Higashi, 1999 ▸)	Numerical (*NUMABS*; Higashi, 1999 ▸)
*T*_min_, *T*_max_	0.896, 0.949	0.925, 0.990
No. of measured, independent and observed [*I* > 2σ(*I*)] reflections	17752, 2789, 2537	12358, 3666, 2755
*R*_int_	0.021	0.122
(sin θ/λ)_max_ (Å^−1^)	0.704	0.704

Refinement		
*R*[*F* ^2^ > 2σ(*F* ^2^)], *wR*(*F* ^2^), *S*	0.030, 0.079, 1.11	0.052, 0.149, 1.01
No. of reflections	2789	3666
No. of parameters	176	197
No. of restraints	8	2
H-atom treatment	H atoms treated by a mixture of independent and constrained refinement	H atoms treated by a mixture of independent and constrained refinement
Δρ_max_, Δρ_min_ (e Å^−3^)	0.54, −0.24	0.52, −0.45

Computer programs: *RAPID-AUTO* (Rigaku, 2006 ▸), *SHELXS97* (Sheldrick, 2008 ▸), *SHELXL2016* (Sheldrick, 2015 ▸), *ORTEP-3 for Windows* (Farrugia, 2012 ▸), *CrystalStructure* (Rigaku, 2010 ▸) and *PLATON* (Spek, 2015 ▸).

## supplementary crystallographic information

### Bis(2-carboxypyridinium) chloranilate dihydrate–bis(pyridinium-2-carboxylate) chloranilic acid dihydrate (1/1) (I) Crystal data

**Table:**

2C_6_H_5.5_NO_2_0.5+·C_6_HCl_2_O_4_^−^·2H_2_O	*F*(000) = 504.00
*M**_r_* = 491.24	*D*_x_ = 1.705 Mg m^−^^3^
Monoclinic, *P*2_1_/*c*	Mo *K*α radiation, λ = 0.71075 Å
*a* = 15.1028 (10) Å	Cell parameters from 15838 reflections
*b* = 3.6851 (3) Å	θ = 3.2–30.0°
*c* = 17.5689 (13) Å	µ = 0.40 mm^−^^1^
β = 101.871 (3)°	*T* = 180 K
*V* = 956.89 (12) Å^3^	Block, brown
*Z* = 2	0.35 × 0.18 × 0.13 mm

### Bis(2-carboxypyridinium) chloranilate dihydrate–bis(pyridinium-2-carboxylate) chloranilic acid dihydrate (1/1) (I) Data collection

**Table:**

Rigaku R-AXIS RAPIDII diffractometer	2537 reflections with *I* > 2σ(*I*)
Detector resolution: 10.000 pixels mm^-1^	*R*_int_ = 0.021
ω scans	θ_max_ = 30.0°, θ_min_ = 3.3°
Absorption correction: numerical (*NUMABS*; Higashi, 1999)	*h* = −21→21
*T*_min_ = 0.896, *T*_max_ = 0.949	*k* = −5→4
17752 measured reflections	*l* = −24→24
2789 independent reflections	

### Bis(2-carboxypyridinium) chloranilate dihydrate–bis(pyridinium-2-carboxylate) chloranilic acid dihydrate (1/1) (I) Refinement

**Table:**

Refinement on *F*^2^	Secondary atom site location: difference Fourier map
*R*[*F*^2^ > 2σ(*F*^2^)] = 0.030	Hydrogen site location: difference Fourier map
*wR*(*F*^2^) = 0.079	H atoms treated by a mixture of independent and constrained refinement
*S* = 1.11	*w* = 1/[σ^2^(*F*_o_^2^) + (0.0445*P*)^2^ + 0.3065*P*] where *P* = (*F*_o_^2^ + 2*F*_c_^2^)/3
2789 reflections	(Δ/σ)_max_ = 0.001
176 parameters	Δρ_max_ = 0.54 e Å^−^^3^
8 restraints	Δρ_min_ = −0.24 e Å^−^^3^
Primary atom site location: structure-invariant direct methods	

### Bis(2-carboxypyridinium) chloranilate dihydrate–bis(pyridinium-2-carboxylate) chloranilic acid dihydrate (1/1) (I) Special details

**Table:**

Geometry. All esds (except the esd in the dihedral angle between two l.s. planes) are estimated using the full covariance matrix. The cell esds are taken into account individually in the estimation of esds in distances, angles and torsion angles; correlations between esds in cell parameters are only used when they are defined by crystal symmetry. An approximate (isotropic) treatment of cell esds is used for estimating esds involving l.s. planes.

### Bis(2-carboxypyridinium) chloranilate dihydrate–bis(pyridinium-2-carboxylate) chloranilic acid dihydrate (1/1) (I) Fractional atomic coordinates and isotropic or equivalent isotropic displacement parameters (Å^2^)

**Table:**

	*x*	*y*	*z*	*U*_iso_*/*U*_eq_	Occ. (<1)
Cl1	0.48813 (2)	0.21752 (7)	0.32764 (2)	0.01728 (8)	
O1	0.64061 (5)	0.6016 (2)	0.43110 (4)	0.02004 (16)	
O2	0.34886 (5)	0.1370 (2)	0.42588 (4)	0.02038 (17)	
H2	0.317 (2)	0.137 (9)	0.4593 (16)	0.031*	0.5
O3	0.06134 (7)	0.3759 (3)	0.54909 (5)	0.0313 (2)	
H3	0.018 (2)	0.445 (12)	0.516 (2)	0.047*	0.5
O4	−0.03494 (6)	0.1653 (3)	0.62162 (5)	0.0303 (2)	
O5A	0.19794 (11)	0.1042 (7)	0.48308 (10)	0.0265 (4)	0.5
H9A	0.1547 (18)	0.038 (11)	0.4493 (18)	0.040*	0.5
H10A	0.2453 (15)	0.043 (11)	0.4682 (18)	0.040*	0.5
O5B	0.20132 (11)	−0.1191 (6)	0.48529 (9)	0.0199 (3)	0.5
H9B	0.1501 (15)	−0.090 (9)	0.4587 (19)	0.030*	0.5
H10B	0.209 (2)	−0.347 (5)	0.4873 (19)	0.030*	0.5
N1	0.19857 (6)	0.0209 (3)	0.63805 (5)	0.01977 (18)	
C1	0.57374 (6)	0.5511 (3)	0.45987 (5)	0.01438 (18)	
C2	0.49294 (7)	0.3688 (3)	0.42158 (5)	0.01476 (18)	
C3	0.42138 (6)	0.3090 (3)	0.45734 (6)	0.01574 (19)	
C4	0.12231 (7)	0.0793 (3)	0.66495 (6)	0.01658 (19)	
C5	0.12193 (7)	0.0166 (3)	0.74206 (6)	0.0198 (2)	
H5	0.068573	0.056090	0.761605	0.024*	
C6	0.20078 (8)	−0.1057 (3)	0.79117 (6)	0.0226 (2)	
H6	0.201710	−0.146932	0.844701	0.027*	
C7	0.27772 (7)	−0.1668 (3)	0.76174 (7)	0.0226 (2)	
H7	0.331694	−0.251819	0.794609	0.027*	
C8	0.27478 (7)	−0.1023 (3)	0.68384 (7)	0.0236 (2)	
H8	0.326860	−0.145258	0.662656	0.028*	
C9	0.04024 (8)	0.2152 (3)	0.60747 (6)	0.0210 (2)	
H1	0.1993 (12)	0.049 (5)	0.5876 (10)	0.039 (4)*	

### Bis(2-carboxypyridinium) chloranilate dihydrate–bis(pyridinium-2-carboxylate) chloranilic acid dihydrate (1/1) (I) Atomic displacement parameters (Å^2^)

**Table:**

	*U*^11^	*U*^22^	*U*^33^	*U*^12^	*U*^13^	*U*^23^
Cl1	0.01956 (13)	0.02040 (13)	0.01184 (12)	−0.00159 (8)	0.00314 (8)	−0.00202 (8)
O1	0.0170 (3)	0.0273 (4)	0.0174 (3)	−0.0029 (3)	0.0071 (3)	−0.0028 (3)
O2	0.0159 (3)	0.0286 (4)	0.0168 (3)	−0.0068 (3)	0.0037 (3)	−0.0038 (3)
O3	0.0340 (5)	0.0363 (5)	0.0200 (4)	−0.0003 (4)	−0.0028 (3)	0.0107 (4)
O4	0.0212 (4)	0.0433 (5)	0.0243 (4)	0.0042 (4)	−0.0003 (3)	0.0001 (4)
O5A	0.0152 (8)	0.0467 (13)	0.0185 (8)	−0.0033 (8)	0.0052 (6)	−0.0080 (8)
O5B	0.0160 (7)	0.0243 (9)	0.0188 (7)	−0.0013 (7)	0.0028 (5)	−0.0010 (7)
N1	0.0231 (4)	0.0215 (4)	0.0160 (4)	0.0002 (4)	0.0071 (3)	−0.0002 (3)
C1	0.0148 (4)	0.0154 (4)	0.0132 (4)	0.0008 (3)	0.0035 (3)	0.0012 (3)
C2	0.0171 (4)	0.0174 (4)	0.0100 (4)	−0.0015 (3)	0.0033 (3)	−0.0007 (3)
C3	0.0147 (4)	0.0186 (5)	0.0137 (4)	0.0012 (4)	0.0025 (3)	0.0021 (3)
C4	0.0177 (4)	0.0163 (4)	0.0151 (4)	−0.0002 (4)	0.0022 (3)	0.0005 (3)
C5	0.0183 (4)	0.0251 (5)	0.0166 (4)	0.0003 (4)	0.0052 (3)	0.0026 (4)
C6	0.0236 (5)	0.0271 (6)	0.0163 (4)	−0.0015 (4)	0.0018 (4)	0.0051 (4)
C7	0.0185 (5)	0.0209 (5)	0.0261 (5)	0.0010 (4)	−0.0009 (4)	0.0032 (4)
C8	0.0197 (5)	0.0245 (5)	0.0281 (5)	0.0025 (4)	0.0083 (4)	−0.0013 (4)
C9	0.0241 (5)	0.0207 (5)	0.0156 (4)	0.0022 (4)	−0.0017 (4)	−0.0013 (4)

### Bis(2-carboxypyridinium) chloranilate dihydrate–bis(pyridinium-2-carboxylate) chloranilic acid dihydrate (1/1) (I) Geometric parameters (Å, º)

**Table:**

Cl1—C2	1.7293 (9)	N1—H1	0.895 (18)
O1—C1	1.2330 (11)	C1—C2	1.4335 (13)
O2—C3	1.2875 (12)	C1—C3^i^	1.5307 (13)
O2—H2	0.830 (18)	C2—C3	1.3751 (13)
O3—C9	1.2801 (14)	C4—C5	1.3754 (13)
O3—H3	0.814 (17)	C4—C9	1.5139 (14)
O4—C9	1.2250 (15)	C5—C6	1.3939 (15)
O5A—H9A	0.824 (18)	C5—H5	0.9500
O5A—H10A	0.841 (18)	C6—C7	1.3840 (16)
O5B—H9B	0.824 (17)	C6—H6	0.9500
O5B—H10B	0.849 (17)	C7—C8	1.3808 (16)
N1—C8	1.3406 (14)	C7—H7	0.9500
N1—C4	1.3492 (13)	C8—H8	0.9500
			
C3—O2—H2	105 (2)	N1—C4—C9	117.38 (9)
C9—O3—H3	115 (3)	C5—C4—C9	122.95 (9)
H9A—O5A—H10A	107 (3)	C4—C5—C6	119.20 (9)
H9B—O5B—H10B	105 (3)	C4—C5—H5	120.4
C8—N1—C4	122.23 (9)	C6—C5—H5	120.4
C8—N1—H1	116.7 (11)	C7—C6—C5	119.83 (10)
C4—N1—H1	120.9 (11)	C7—C6—H6	120.1
O1—C1—C2	124.63 (9)	C5—C6—H6	120.1
O1—C1—C3^i^	117.08 (9)	C8—C7—C6	118.95 (10)
C2—C1—C3^i^	118.29 (8)	C8—C7—H7	120.5
C3—C2—C1	122.26 (8)	C6—C7—H7	120.5
C3—C2—Cl1	120.12 (8)	N1—C8—C7	120.11 (10)
C1—C2—Cl1	117.62 (7)	N1—C8—H8	119.9
O2—C3—C2	124.19 (9)	C7—C8—H8	119.9
O2—C3—C1^i^	116.38 (8)	O4—C9—O3	128.74 (11)
C2—C3—C1^i^	119.43 (8)	O4—C9—C4	118.74 (10)
N1—C4—C5	119.67 (9)	O3—C9—C4	112.51 (10)
			
O1—C1—C2—C3	177.79 (10)	N1—C4—C5—C6	0.18 (17)
C3^i^—C1—C2—C3	−1.60 (16)	C9—C4—C5—C6	−179.46 (10)
O1—C1—C2—Cl1	−1.39 (14)	C4—C5—C6—C7	−0.86 (17)
C3^i^—C1—C2—Cl1	179.22 (7)	C5—C6—C7—C8	0.46 (18)
C1—C2—C3—O2	−177.81 (10)	C4—N1—C8—C7	−1.34 (17)
Cl1—C2—C3—O2	1.35 (15)	C6—C7—C8—N1	0.62 (18)
C1—C2—C3—C1^i^	1.61 (16)	N1—C4—C9—O4	157.55 (11)
Cl1—C2—C3—C1^i^	−179.22 (7)	C5—C4—C9—O4	−22.81 (16)
C8—N1—C4—C5	0.93 (17)	N1—C4—C9—O3	−23.17 (14)
C8—N1—C4—C9	−179.41 (10)	C5—C4—C9—O3	156.48 (11)

Symmetry code: (i) −*x*+1, −*y*+1, −*z*+1.

### Bis(2-carboxypyridinium) chloranilate dihydrate–bis(pyridinium-2-carboxylate) chloranilic acid dihydrate (1/1) (I) Hydrogen-bond geometry (Å, º)

**Table:**

*D*—H···*A*	*D*—H	H···*A*	*D*···*A*	*D*—H···*A*
N1—H1···O5*A*	0.895 (17)	1.843 (17)	2.7380 (19)	179 (2)
N1—H1···O5*B*	0.895 (17)	1.907 (17)	2.7417 (18)	154.4 (17)
O2—H2···O5*B*	0.83 (3)	2.12 (3)	2.8111 (19)	141 (3)
O3—H3···O3^ii^	0.82 (3)	1.62 (4)	2.4352 (14)	174 (4)
O5*A*—H9*A*···O4^iii^	0.82 (3)	2.11 (3)	2.927 (2)	170 (3)
O5*B*—H9*B*···O4^iii^	0.82 (3)	2.02 (3)	2.8159 (19)	162 (3)
O5*A*—H10*A*···O2	0.84 (3)	1.90 (3)	2.6762 (19)	154 (4)
O5*B*—H10*B*···O1^iv^	0.85 (2)	2.60 (3)	3.095 (2)	119 (2)
C8—H8···Cl1^iv^	0.95	2.78	3.6524 (12)	154
C8—H8···O1^iv^	0.95	2.47	3.1871 (14)	132

Symmetry codes: (ii) −*x*, −*y*+1, −*z*+1; (iii) −*x*, −*y*, −*z*+1; (iv) −*x*+1, −*y*, −*z*+1.

### chloranilic acid–2-carboxyquinoline (1/2) dihydrate (II) Crystal data

**Table:**

2C_10_H_7_NO_2_·C_6_H_2_Cl_2_O_4_·2H_2_O	*Z* = 1
*M**_r_* = 591.36	*F*(000) = 304.00
Triclinic, *P*1	*D*_x_ = 1.565 Mg m^−^^3^
Hall symbol: -P 1	Mo *K*α radiation, λ = 0.71075 Å
*a* = 4.4745 (2) Å	Cell parameters from 9714 reflections
*b* = 10.5448 (8) Å	θ = 3.0–30.1°
*c* = 13.6111 (6) Å	µ = 0.32 mm^−^^1^
α = 96.652 (4)°	*T* = 200 K
β = 94.109 (3)°	Platelet, brown
γ = 99.009 (4)°	0.41 × 0.21 × 0.03 mm
*V* = 627.38 (6) Å^3^	

### chloranilic acid–2-carboxyquinoline (1/2) dihydrate (II) Data collection

**Table:**

Rigaku R-AXIS RAPIDII diffractometer	2755 reflections with *I* > 2σ(*I*)
Detector resolution: 10.000 pixels mm^-1^	*R*_int_ = 0.122
ω scans	θ_max_ = 30.0°, θ_min_ = 3.0°
Absorption correction: numerical (*NUMABS*; Higashi, 1999)	*h* = −6→6
*T*_min_ = 0.925, *T*_max_ = 0.990	*k* = −14→14
12358 measured reflections	*l* = −19→19
3666 independent reflections	

### chloranilic acid–2-carboxyquinoline (1/2) dihydrate (II) Refinement

**Table:**

Refinement on *F*^2^	Secondary atom site location: difference Fourier map
*R*[*F*^2^ > 2σ(*F*^2^)] = 0.052	Hydrogen site location: inferred from neighbouring sites
*wR*(*F*^2^) = 0.149	H atoms treated by a mixture of independent and constrained refinement
*S* = 1.00	*w* = 1/[σ^2^(*F*_o_^2^) + (0.0879*P*)^2^] where *P* = (*F*_o_^2^ + 2*F*_c_^2^)/3
3666 reflections	(Δ/σ)_max_ < 0.001
197 parameters	Δρ_max_ = 0.52 e Å^−^^3^
2 restraints	Δρ_min_ = −0.45 e Å^−^^3^
Primary atom site location: structure-invariant direct methods	

### chloranilic acid–2-carboxyquinoline (1/2) dihydrate (II) Special details

**Table:**

Geometry. All esds (except the esd in the dihedral angle between two l.s. planes) are estimated using the full covariance matrix. The cell esds are taken into account individually in the estimation of esds in distances, angles and torsion angles; correlations between esds in cell parameters are only used when they are defined by crystal symmetry. An approximate (isotropic) treatment of cell esds is used for estimating esds involving l.s. planes.

### chloranilic acid–2-carboxyquinoline (1/2) dihydrate (II) Fractional atomic coordinates and isotropic or equivalent isotropic displacement parameters (Å^2^)

**Table:**

	*x*	*y*	*z*	*U*_iso_*/*U*_eq_	
Cl1	0.49390 (9)	0.23664 (4)	−0.02842 (3)	0.04300 (16)	
O1	0.0319 (3)	0.08081 (12)	−0.17653 (8)	0.0426 (3)	
O2	0.4150 (3)	0.11710 (11)	0.15510 (9)	0.0391 (3)	
O3	−0.1769 (3)	0.36216 (11)	0.47121 (9)	0.0372 (3)	
O4	−0.0712 (3)	0.16160 (11)	0.43604 (9)	0.0425 (3)	
O5	0.3938 (3)	0.07471 (12)	0.33231 (9)	0.0406 (3)	
N1	0.3233 (3)	0.41970 (12)	0.60390 (9)	0.0298 (3)	
C1	0.0207 (3)	0.04635 (14)	−0.09469 (11)	0.0317 (3)	
C2	0.2276 (3)	0.10762 (14)	−0.00985 (11)	0.0320 (3)	
C3	0.2197 (3)	0.06514 (14)	0.07971 (11)	0.0313 (3)	
C4	0.2151 (3)	0.29703 (14)	0.56909 (11)	0.0305 (3)	
C5	0.3274 (3)	0.19702 (15)	0.61140 (11)	0.0336 (3)	
H5	0.250293	0.109195	0.586437	0.040*	
C6	0.5493 (4)	0.22680 (15)	0.68906 (12)	0.0342 (3)	
H6	0.622423	0.159293	0.719133	0.041*	
C7	0.6694 (3)	0.35659 (14)	0.72449 (11)	0.0312 (3)	
C8	0.9050 (4)	0.39332 (16)	0.80217 (12)	0.0363 (3)	
H8	0.985438	0.328869	0.834065	0.044*	
C9	1.0170 (4)	0.52094 (18)	0.83147 (13)	0.0411 (4)	
H9	1.175836	0.545100	0.883608	0.049*	
C10	0.8980 (4)	0.61764 (17)	0.78463 (13)	0.0418 (4)	
H10	0.980263	0.706066	0.805678	0.050*	
C11	0.6677 (4)	0.58722 (15)	0.70983 (13)	0.0372 (3)	
H11	0.587483	0.653070	0.679646	0.045*	
C12	0.5532 (3)	0.45558 (14)	0.67897 (10)	0.0294 (3)	
C13	−0.0333 (3)	0.27243 (14)	0.48397 (11)	0.0315 (3)	
H1	0.252 (5)	0.481 (2)	0.5760 (17)	0.055 (6)*	
H2	0.359 (7)	0.083 (3)	0.216 (2)	0.092 (10)*	
H3	0.255 (4)	0.086 (2)	0.3667 (15)	0.054 (6)*	
H4	0.560 (4)	0.111 (2)	0.3651 (17)	0.063 (7)*	

### chloranilic acid–2-carboxyquinoline (1/2) dihydrate (II) Atomic displacement parameters (Å^2^)

**Table:**

	*U*^11^	*U*^22^	*U*^33^	*U*^12^	*U*^13^	*U*^23^
Cl1	0.0402 (2)	0.0380 (2)	0.0465 (2)	−0.00570 (16)	−0.00095 (18)	0.00725 (17)
O1	0.0451 (7)	0.0452 (7)	0.0354 (6)	0.0008 (5)	−0.0026 (5)	0.0093 (5)
O2	0.0388 (6)	0.0380 (6)	0.0359 (6)	−0.0005 (5)	−0.0079 (5)	0.0012 (4)
O3	0.0376 (6)	0.0319 (6)	0.0427 (6)	0.0068 (4)	−0.0017 (5)	0.0089 (4)
O4	0.0403 (6)	0.0368 (6)	0.0471 (6)	0.0091 (5)	−0.0071 (5)	−0.0052 (5)
O5	0.0400 (7)	0.0419 (7)	0.0357 (6)	0.0047 (5)	−0.0066 (5)	−0.0029 (5)
N1	0.0302 (6)	0.0269 (6)	0.0326 (6)	0.0053 (5)	0.0020 (5)	0.0055 (5)
C1	0.0292 (7)	0.0304 (7)	0.0353 (7)	0.0061 (5)	−0.0004 (6)	0.0039 (6)
C2	0.0287 (7)	0.0274 (7)	0.0384 (7)	0.0022 (5)	−0.0010 (6)	0.0032 (5)
C3	0.0298 (7)	0.0278 (7)	0.0354 (7)	0.0064 (5)	−0.0016 (6)	0.0007 (5)
C4	0.0297 (7)	0.0291 (7)	0.0330 (7)	0.0046 (5)	0.0044 (6)	0.0045 (5)
C5	0.0347 (7)	0.0273 (7)	0.0384 (7)	0.0043 (5)	0.0005 (6)	0.0051 (5)
C6	0.0357 (7)	0.0301 (7)	0.0378 (7)	0.0059 (6)	0.0020 (6)	0.0089 (6)
C7	0.0299 (7)	0.0315 (7)	0.0325 (7)	0.0047 (5)	0.0039 (6)	0.0054 (5)
C8	0.0347 (7)	0.0388 (8)	0.0353 (7)	0.0063 (6)	0.0011 (6)	0.0060 (6)
C9	0.0358 (8)	0.0453 (9)	0.0382 (8)	0.0033 (7)	−0.0036 (7)	−0.0030 (7)
C10	0.0402 (8)	0.0329 (8)	0.0484 (9)	0.0026 (6)	0.0006 (7)	−0.0046 (7)
C11	0.0376 (8)	0.0289 (7)	0.0443 (8)	0.0061 (6)	0.0027 (7)	0.0014 (6)
C12	0.0281 (6)	0.0287 (7)	0.0311 (6)	0.0044 (5)	0.0042 (6)	0.0021 (5)
C13	0.0293 (7)	0.0311 (7)	0.0339 (7)	0.0037 (5)	0.0024 (6)	0.0058 (5)

### chloranilic acid–2-carboxyquinoline (1/2) dihydrate (II) Geometric parameters (Å, º)

**Table:**

Cl1—C2	1.7174 (15)	C4—C13	1.517 (2)
O1—C1	1.2130 (18)	C5—C6	1.370 (2)
O2—C3	1.3070 (17)	C5—H5	0.9500
O2—H2	0.98 (3)	C6—C7	1.405 (2)
O3—C13	1.2453 (18)	C6—H6	0.9500
O4—C13	1.2512 (18)	C7—C8	1.413 (2)
O5—H3	0.821 (16)	C7—C12	1.420 (2)
O5—H4	0.848 (16)	C8—C9	1.363 (2)
N1—C4	1.3270 (19)	C8—H8	0.9500
N1—C12	1.3722 (19)	C9—C10	1.415 (3)
N1—H1	0.88 (2)	C9—H9	0.9500
C1—C2	1.447 (2)	C10—C11	1.368 (2)
C1—C3^i^	1.507 (2)	C10—H10	0.9500
C2—C3	1.348 (2)	C11—C12	1.406 (2)
C4—C5	1.401 (2)	C11—H11	0.9500
			
C3—O2—H2	111.3 (18)	C7—C6—H6	119.8
H3—O5—H4	108 (2)	C6—C7—C8	123.01 (14)
C4—N1—C12	122.93 (13)	C6—C7—C12	118.61 (13)
C4—N1—H1	119.1 (16)	C8—C7—C12	118.37 (14)
C12—N1—H1	117.9 (16)	C9—C8—C7	120.18 (15)
O1—C1—C2	123.09 (14)	C9—C8—H8	119.9
O1—C1—C3^i^	118.94 (13)	C7—C8—H8	119.9
C2—C1—C3^i^	117.97 (13)	C8—C9—C10	120.32 (15)
C3—C2—C1	122.39 (13)	C8—C9—H9	119.8
C3—C2—Cl1	120.62 (11)	C10—C9—H9	119.8
C1—C2—Cl1	116.98 (11)	C11—C10—C9	121.78 (15)
O2—C3—C2	122.30 (14)	C11—C10—H10	119.1
O2—C3—C1^i^	118.13 (13)	C9—C10—H10	119.1
C2—C3—C1^i^	119.57 (12)	C10—C11—C12	118.00 (15)
N1—C4—C5	120.22 (13)	C10—C11—H11	121.0
N1—C4—C13	116.94 (13)	C12—C11—H11	121.0
C5—C4—C13	122.84 (13)	N1—C12—C11	120.39 (13)
C6—C5—C4	119.50 (14)	N1—C12—C7	118.27 (13)
C6—C5—H5	120.2	C11—C12—C7	121.34 (14)
C4—C5—H5	120.2	O3—C13—O4	128.04 (14)
C5—C6—C7	120.39 (14)	O3—C13—C4	117.19 (13)
C5—C6—H6	119.8	O4—C13—C4	114.76 (13)
			
O1—C1—C2—C3	−177.43 (15)	C12—C7—C8—C9	−0.4 (2)
C3^i^—C1—C2—C3	2.8 (2)	C7—C8—C9—C10	0.2 (3)
O1—C1—C2—Cl1	1.2 (2)	C8—C9—C10—C11	0.5 (3)
C3^i^—C1—C2—Cl1	−178.60 (10)	C9—C10—C11—C12	−1.0 (3)
C1—C2—C3—O2	177.00 (13)	C4—N1—C12—C11	177.18 (14)
Cl1—C2—C3—O2	−1.6 (2)	C4—N1—C12—C7	−3.2 (2)
C1—C2—C3—C1^i^	−2.8 (2)	C10—C11—C12—N1	−179.65 (14)
Cl1—C2—C3—C1^i^	178.61 (10)	C10—C11—C12—C7	0.7 (2)
C12—N1—C4—C5	2.4 (2)	C6—C7—C12—N1	1.5 (2)
C12—N1—C4—C13	−178.34 (12)	C8—C7—C12—N1	−179.66 (13)
N1—C4—C5—C6	0.1 (2)	C6—C7—C12—C11	−178.84 (14)
C13—C4—C5—C6	−179.12 (14)	C8—C7—C12—C11	0.0 (2)
C4—C5—C6—C7	−1.6 (2)	N1—C4—C13—O3	−19.8 (2)
C5—C6—C7—C8	−177.95 (14)	C5—C4—C13—O3	159.47 (14)
C5—C6—C7—C12	0.8 (2)	N1—C4—C13—O4	161.50 (14)
C6—C7—C8—C9	178.31 (16)	C5—C4—C13—O4	−19.3 (2)

Symmetry code: (i) −*x*, −*y*, −*z*.

### chloranilic acid–2-carboxyquinoline (1/2) dihydrate (II) Hydrogen-bond geometry (Å, º)

**Table:**

*D*—H···*A*	*D*—H	H···*A*	*D*···*A*	*D*—H···*A*
N1—H1···O3^ii^	0.88 (2)	1.91 (2)	2.7724 (17)	167 (2)
O2—H2···O5	0.98 (3)	1.59 (3)	2.5092 (17)	155 (3)
O5—H3···O4	0.82 (2)	2.01 (2)	2.8072 (19)	164 (2)
O5—H4···O4^iii^	0.85 (2)	1.82 (2)	2.6632 (19)	171 (2)
C6—H6···O1^iv^	0.95	2.54	3.392 (2)	150
C6—H6···O5^v^	0.95	2.47	3.211 (2)	134

Symmetry codes: (ii) −*x*, −*y*+1, −*z*+1; (iii) *x*+1, *y*, *z*; (iv) *x*+1, *y*, *z*+1; (v) −*x*+1, −*y*, −*z*+1.
